# GENETICS 2023 Reviewer Index

**DOI:** 10.1093/genetics/iyad193

**Published:** 2023-12-01

Below is a list of individuals who have reviewed manuscripts for *GENETICS* during the preparation of Volumes 223, 224, and 225 (2023). The aim of GENETICS is to communicate significant research. To succeed, we must recognize what is significant, making sure the presentation is accurate, unambiguous, and easily readable. For this, we depend heavily upon our reviewers, who perform this indispensable service without reward. The quality of the journal strongly reflects the quality of their efforts. We apologize to any reviewer whose name has been inadvertently omitted, and we are sincerely grateful to all.

Abalde-Atristain, Leire

Abbott, Allison

Abdelrahman, Mostafa

Ables, Elizabeth

Abu Awad, Diala

Achaz, Guillaume

Ackerman, Sarah

Ackert, Lloyd

Ackert-Bicknell, Cheryl

Ackley, Brian

Adhikari, Kiran

Affolter, Markus

Afzal, Mohammad

Agashe, Deepa

Agren, Arvid

Ahmed-Braimah, Yasir

Ahnert, Sebastian

Ahringer, Julie

Aiden, Erez

Aigaki, Toshiro

Ainouche, Malika

Aklilu, Behailu

Alani, Eric

Albert, Jeffrey

Albertson, Craig

Aldinger, Kimberly

Alfonzo, Juan

Allada, Ravi

Allshire, Robin

Amodeo, Amanda

Amoutzias, Grigoris

Amundsen, Keenan

Amundson, Kirk

Andergassen, Daniel

Andersen, Erik

Anderson, Eric

Anderson, Matthew

Andolfatto, Peter

Andrews, Brenda

Andrianopoulos, Alex

Anholt, Robert

Ansari, Athar

Aquadro, Charles

Aquino Nunez, Wendy

Araújo, Sofia

Argueso, Juan Lucas

Arkov, Alexey

Arnone, Maria Ina

Arnoult, Nausica

Arribere, Joshua

Artal-Sanz, Marta

Asahina, Kenta

Askjaer, Peter

Assié, Adrien

Atkinson, Elizabeth

Aubier, Thomas

Audet, Céline

Auld, Vanessa

Aurell, Erik

Auwrex, Johan

Avadhanam, Siddharth

Aydin, Selcan

Aylor, David

Ayroles, Julien

Azodi, Christina

Azpiazu, Natalia

Azzalin, Claus

Baake, Ellen

Bach, Erika

Bachtrog, Doris

Badet, Thomas

Baehrecke, Eric

Baetz, Kristin

Bähler, Jürg

Bai, Xiaofei

Bainton, Roland

Baker, EmilyClare

Balding, David

Baldock, Clair

Balint-Kurti, Peter

Ballerini, Evangeline

Banerjee, Surya

Banerjee, Utpal

Banerjee-Basu, Sharmila

Bank, Claudia

Bankevich, Anton

Banse, Stephen

Barbash, Daniel

Barclay, Jeff

Bardin, Allison

Barish, Scott

Barkai, Naama

Barker, Bridget

Barr, Maureen

Barral, Yves

Barrios, Arantza

Bartley, Devin

Barton, Lacy

Bataillon, Thomas

Bateman, Jack

Bateman, Alex

Baucom, Regina

Bauer, Johannes

Baugh, L.

Bayer, Phillip

Becker, Doreen

Bedalov, Antonio

Beitel, Greg

Bejsovec, Amy

Ben-Shahar, Yehuda

Benanti, Jennifer

Bendixsen, Devin

Bensasson, Douda

Benton, Richard

Berardi, Andrea

Berardini, Tanya

Berdan, Emma

Berg, Jeremy

Berman, Judith

Bernardo, Rex

Bernstein, Sanford

Bernstein, Richard

Bertram, Jason

Bertuch, Alison

Besse, Florence

Bessereau, Jean-Louis

Bhat, Umer

Bhattacharya, Abhishek

Bi, Erfei

Bickel, Sharon

Biddanda, Arjun

Bielas, Jason

Bielinsky, Anja

Bier, Ethan

Bierne, Nicolas

Biesecker, Les

Biggins, Sue

Bijma, Piter

Bilder, David

Billmyre, Katie

Birchler, James

Birkner, Matthias

Birol, Inanç

Bischoff, Joyce

Bishop, Douglas

Biteau, Benoit

Blackman, Benjamin

Blackmon, Heath

Blankenship, Todd

Blau, Justin

Bloom, Kerry

Blumenstiel, Justin

Boatwright, J.

Bochman, Matthew

Bock, Christoph

Boeke, Jef

Boitard, Simon

Bonetti, Diego

Borgen, Melissa

Borjon, Lydia

Borst, Alexander

Bose, Aritra

Bosworth, Brian

Bouchet, Sophie

Boulin, Thomas

Bouwman, Aniek

Bracewell, Ryan

Bradburd, Gideon

Bradbury, Peter

Branco, Sara

Brand, Alexandra

Brandvain, Yaniv

Brar, Gloria

Brash, Douglas

Bray, Sarah

Brennecke, Julius

Brenner, Charles

Breslin, Paul

Briggs, Steven

Brill, Julie

Bringmann, Henrik

Brockmann, Gudrun

Broman, Karl

Brow, David

Brown, Kevin

Brown, Alistair

Browning, Sharon

Bruford, Elspeth

Bruno, Vincent

Brush, George

Bruzzone, Maria

Bryan, Glenn

Bryan, Tracy

Bryantsev, Anton

Bubnell, Jaclyn

Buchler, Nicolas

Buck, Jochen

Buckley, Katherine

Buerger, Reinhard

Buffalo, Vince

Buffone, Mariano

Bühler, Marc

Bukhari, Hassan

Bult, Carol

Bunk, Boyke

Buratowski, Stephen

Burchard, Esteban

Burke, Brian

Burnett, Angie

Burri, Reto

Busch, Emanuel

Bustillo, Maria

Bustos-Korts, Daniela

Busturia, Ana

Buszczak, Michael

Butler, Geraldine

Caballero, Armando

Cabrera, Claudia

Cadney, Marcell

Cagan, Alex

Cai, Haoran

Calus, Mario

Capel, Blanche

Capra, John

Careau, Vincent

Carlborg, Örjan

Carleton, Karen

Carmi, Shai

Carputo, Domenico

Carrera, Ines

Carthew, Richard

Casal, Jorge

Casale, Assunta Maria

Castaneda-Bueno, Maria

Castello, Alfredo

Castiglione, Gianni

Castro, Victoria

Catalán, Ana

Cates, Kitra

Cavalieri, Duccio

Cavalieri, Duccio

Cecere, Germano

Cejka, Petr

Celen, Irem

Chang, Michael

Chang, Joseph

Chappidi, Sruthi

Charlesworth, Brian

Chatterjee, Nimrat

Chaudhari, Karina

Chee, Peng

Chen, Li

Chen, Liang

Chen, Hua

Chen, Kaifu

Chen, Shifu

Chen, Jiakun

Chen, Han

Chen, Nancy

Chen, Jingxian

Chen, Hsiang-Cheng

Chenoweth, Stephen

Cheung, Alan

Cheverud, James

Chevin, Luis-Miguel

Chiang, Charleston

Choe, Keith

Chung, Bon-chu

Church, Michael

Civetta, Alberto

Cleaver, Ondine

Coate, Jeremy

Cobb, Jennifer

Cochella, Luisa

Cohen, Barak

Cole, John

Coleman-Derr, Devin

Colodner, Kenneth

Colot, Vincent

Comai, Luca

Comeron, Josep

Comi, Anne

Conaty, Warren

Conesa, Ana

Conklin, Phillip

Connallon, Tim

Conradt, Barbara

Cook, Kevin

Corbett, Anita

Corbett-Detig, Russell

Cordell, Heather

Corden, Jeffry

Cormack, Brendan

Corty, Megan

Costa, Vitor

Côté, Jacques

Counterman, Brian

Courcelle, Justin

Courret, Cécile

Coutinho-Budd, Jaeda

Cox, Robert

Cox, Rachel

Cram, Erin

Cramer, Robert

Crespel, Amelie

Crisanti, Andrea

Cromie, Gareth

Crown, Nicole

Cubillos, Francisco

Cuervo, Anna Maria

Cui, Yuehua

Cuninngham, Julie

Cutter, Asher

Cvijovic, Ivana

D'Hont, Angelique

Daetwyler, Hans

Daignan-Fornier, Bertrand

Dammermann, Alexander

Davis, Ilan

Dawe, R.

De Bono, Mario

De Cuevas, Margaret

De Folter, Stefan

De Leon, Natalia

De los Campos, Gustavo

De Manuel Montero, Marc

De Massy, Bernard

De Meaux, Juliette

De Oliveira Rocha, Raquel

De Pitta, Cristiano

De villemereuil, Pierre

Debes, Paul Vincent

Degnan, James

DeJong, Hans

DeJong-Lambert, William

Dekens, Leonard

Dekkers, Jack

Delattre, Marie

Delneri, Daniela

DeLuca, Steven

Deng, Yun

Deng, Wu-Min

Dent, Sharon

Desai, Michael

Desbois, Muriel

Desplan, Claude

Devi, Archana

Diaz-Papkovich, Alex

Dick, Frederick

Dickinson, Daniel

Dieckmann, Carol

Dilkes, Brian

DiMario, Patrick

Dini-Andreote, Francisco

Do, Eunsoo

Doebley, John

Doetsch, Paul

Dolinski, Kara

Donaldson, Anne

Donczew, Rafal

Dondi, Cristiana

Dos Santos Carvalho, Livia

Douches, David

Drews, Gary

Driscoll, Monica

Drown, Melissa

Dudbridge, Frank

Dudley, Aimee

Duenk, Pascal

Duester, Gregg

Dumont, Beth

Dunham, Maitreya

Durand, Dannie

Durbin, Richard

Duronio, Robert

Dutheil, Julien

Dutta, Debdeep

Dweck, Hany

Dyson, Nicholas

Eagen, Kyle

Ebou, Anicet

Eden, Emily

Ehrenreich, Ian

Eisenmann, David

Ekker, Stephen

Ekwall, Karl

Elgin, Sarah

Ellis, Ronald

Elsen, Jean-Michel

Emerson, Soren

Engel, Stacia

Ephrussi, Anne

Erbe, Malena

Erickson, James

Essner, Jeffrey

Everman, Elizabeth

Ewen-Campen, Ben

Fairbanks, Daniel

Fang, Junnan

Fang, David

Fanti, Laura

Fasullo, Michael

Félix, Marie-Anne

Fellmeth, Jessica

Fernandes, Joyce

Ferree, Patrick

Fijarczyk, Anna

Finucane, Hilary

Fisher, Malcolm

Flavell, Steven

Foiani, Marco

Formosa, Tim

Forsburg, Susan

Fortes-Lima, Cesar

Fortwendel, Jarrod

Fournier, Elisabeth

Fowler, John

Fox, Donald

Fox, Catherine

Fraïsse, Christelle

Francis, Michael

Francois, Olivier

Frank, C.

Fraser, Hunter

Fraser, Abigail

Frayling, Tim

Freda, Philip

Freeman, Marc

Frenoy, Antoine

Freudenreich, Catherine

Freund, Fabian

Frøkjær-Jensen, Christian

Frolov, Maxim

Frost, Bess

Frydman, Horacio

Fujita, Naonobu

Fuller, Margaret

Fung, Jennifer

Furlong, Eileen

Futcher, Bruce

Gadadhar, Sudarshan

Gage, Joseph

Gaggiotti, Oscar

Galande, Sanjeev

Gáliková, Martina

Gallagher, Joseph

Gallicchio, Lorenzo

Galupa, Rafael

Ganguly, Anindya

Gao, Ziyue

Gao, Zhonghua

Garant, Dany

Garcia, David

Garland, Theodore

Garriga, Gian

Gartenberg, Marc

Gasser, Susan

Gatti, Daniel

Gaut, Brandon

Gautier, Mathieu

Gavis, Elizabeth

Geelen, Danny

Geiler-Samerotte, Kerry

Geisbrecht, Erika

Gemmell, Patrick

Gent, Jonathan

Gentile, Gabrielle

Gerard, David

Gerrish, Philip

Gerton, Jennifer

Gfeller, Valentin

Ghabrial, Amin

Gharrett, Anthony

Ghose, Piya

Giangrande, Angela

Gibbs, Allen

Gibert, Jean-Michel

Gibson, Greg

Gignoux, Chris

Gignoux, Christopher

Gilbert, Wendy

Gill, Dipender

Giraud, Tatiana

Gitler, Aaron

Gjerde, Bjarne

Gladieux, Pierre

Glémin, Sylvain

Glover, David

Goddard, Matthew

Godenschwege, Tanja

Goff, Stephen

Golden, Andy

Goldman, Gustavo

Golic, Kent

Gomes, José Eduardo

Gomes, Edgar

Gomez-Diaz, Carolina

Gompert, Zachariah

Gomulkiewicz, Richard

Gong, Boying

Gong, Boying

Gonsalvez, Graydon

Gonzalez, Oscar

Gonzalez, Josefa

Gonzalo, Adrian

Good, Benjamin

Good, Jeffrey

Goodman, Miriam

Goodrich, Justin

Goodwin, Stephen

Gordo, Isabel

Gorelick, Daniel

Gorjanc, Gregor

Gotenstein, Jennifer

Gotting, Kirsten

Goudet, Jérôme

Gould, Alex

Gower, Graham

Gramazio, Pietro

Gravel, Simon

Greaves, Ian

Green, Erin

Green, Richard

Greenberg, Maxim

Greenberg, Miriam

Greenstein, David

Greenwood, Celia

Gresham, David

Grishok, Alla

Grosshans, Joerg

Grosshans, Helge

Grossniklaus, Ueli

Gruber, Stephan

Gu, Sam

Gu, April

Guerrero, Rafael

Guindon, Stephane

Gullerova, Monika

Gunsalus, Kris

Guo, Bin

Guo, Huihong

Gupta, Bhagwati

Gupta, Mohan

Gurevich, Alexey

Gusev, Alexander

Gutenkunst, Ryan

Haag, Eric

Haas, Hubertus

Hahn, Steven

Hake, Sarah

Hallem, Elissa

Haltiwanger, Robert

Hammell, Christopher

Hammond, Thomas

Hammond-Kosack, Kim

Han, Kyung-An

Hanna, Courtney

Hao, Yue

Hardin, Paul

Harris, Steven

Hart, Anne

Hartenstein, Volker

Hartfield, Matthew

Hartl, Daniel

Hartmann, Fanny

Hase, Yoshihiro

Hastings, Janna

Hawley, R. Scott

Haynes, Cole

Heasley, Lydia

Heiman, Maxwell

Helfand, Stephen

Hemani, Gibran

Henderson, Ian

Henke, Katrin

Henry, Isabelle

Hentze, Matthias

Hermjakob, Henning

Heslop-Harrison, Pat

Hevner, Robert

Hinman, Veronica

Hisamoto, Naoki

Ho, Wei-Chin

Hobert, Oliver

Hobolth, Asger

Hochstrasser, Mark

Hochwagen, Andreas

Hoekstra, Hopi

Hofmanová, Zuzana

Holland, James

Hollingsworth, Nancy

Hombauer, Hans

Hong-Wen, Tang

Honigberg, Michael

Hopper, Anita

Hopyan, Sevan

Horb, Marko

Horne-Badovinac, Sally

Hosaka, Kazuyoshi

Houben, Andreas

Houry, Walid

Housworth, Elizabeth

Howe, Douglas

Hsieh, Yu-Ying Phoebe

Hu, Yiming

Huang, Wen

Huang, Jinling

Hubbard, E. Jane

Hubmacher, Dirk

Hucklehoven, Ralph

Hudry, Bruno

Huerta-Sánchez, Emilia

Hufford, Matthew

Hulse-Kemp, Amanda

Humphrey, Jay

Hunter, Neil

Hurd, Thomas

Hutchinson, John

Hutter, Harald

Huynh, Jean-Rene

Idnurm, Alexander

Igaki, Tatsushi

Im, Hae Kyung

Immler, Simone

Inoue, Naokazu

Ioannou, Maria

Iqbal, Hira

Irish, Vivian

Islam, Mirazul

Izawa, Takeshi

Izquierdo, Paulo

Jackman, Jane

Jackman, Shaun

Jagla, Teresa

Jain, Kavita

Jakubek, Yasminka

James, Anthony

Jannink, Jean-Luc

Jannink, Jean-Luc

Janzen, Thijs

Jaramillo-Lambert, Aimee

Jareczek, Josef

Jay, Flora

Jean, Steve

Jenczewski, Eric

Jenkins, Paul

Jiang, Yong

Jiang, Lan

Jiang, Jin

Jighly, Abdulqader

Jin, Xin

Jin, Yishi

Johannesson, Hanna

Johnson, Alexander

Johnson, Mark

Johnsson, Martin

Johnston, Laura

Johri, Parul

Joiner, William

Jones, Richard

Jones, Matt

Jordan, David

Jordan, Rebecca

Jordan, I.

Jorgensen, Erik

Josephs, Emily

Joukhadar, Reem

Juhász, Gabor

Kachroo, Aashiq

Kadosh, David

Kai, Toshie

Kalderon, Daniel

Kanca, Oguz

Kane, Nolan

Kang, Hyun Min

Kang, Si-Yong

Kaplan, Joshua

Kapun, Martin

Karasik, David

Karp, Xantha

Karpen, Gary

Kashtelyan, Cooper

Kassir, Yona

Kassis, Judith

Kasturi, Prasad

Kater, Martin

Kaufman, Thomas

Kaufman, Kerstin

Kaufman, Paul

Kaun, Karla

Kawecki, Tadeusz

Kayondo, Siraj

Ke, Wenfan

Keele, Gregory

Keeling, Patrick

Keene, Alex

Keinan, Alon

Keleman, Krystyna

Kelleher, Erin

Kelliher, Christina

Kellogg, Elizabeth

Kelly, John

Kelsey, Gavin

Kennedy, Scott

Keogh, Michael

Kerr, Benjamin

Kerscher, Oliver

Kiel, Douglas

Kiger, Amy

Kim, Kyuhyung

Kim, Nayun

Kim, John

Kim-Hellmuth, Sarah

King, Elizabeth

Kirkpatrick, Mark

Kirkpatrick, Mark

Kishore, Ranjana

Kitamura, Satoshi

Kitaoka, Maiko

Klaembt, Christian

Klein, Hannah

Kliebenstein, Daniel

Kloock, Anke

Kobayashi, Hisato

Koh, Kyunghee

Konwar, Chandrika

Koornneef, Maarten

Kopecky, David

Koren, Sergey

Kornberg, Tom

Korunes, Katharine

Koshland, Douglas

Kost, Luise

Koushika, Sandhya

Kozak, Genevieve

Kramer, Helmut

Krebs, Morten

Kristensen, Torsten Nygaard

Kronenberg, Zev

Kronstad, James

Krug, Joachim

Kruuk, Loeske

Kubatko, Laura

Kuchler, Karl

Kühnlein, Ronald

Kumar, Tarun

Kumar, Sandeep

Kupiec, Martin

Kuraparthy, Vasu

Kuroda, Mitzi

Kuzminov, Andrei

Kwak, Soo Heon

Kwitek, Anne

Kyriazis, Chris

Labib, Karim

Lacin, Haluk

Lackner, Laura

Ladejobi, Funmi

Lambert, Amaury

Lambert, Amaury

Landry, Christian

Lang, Gregory

Langley, Charles

Langner, Thorsten

Larson, Erica

Lascoux, Martin

Lasko, Paul

Lasky, Jesse

Law, Julie

Lawson, Nathan

Lawson, Heather

Le Rouzic, Arnaud

Lear, Bridget

Leblois, Raphaël

Lee, Yuh Chwen

Lee, Kwan Yin

Lee, Sang

Lee, Soo

Lee, Seunggeun

Lee, I-Ju

Lee, Daehan

Legarra, Andres

Lehmann, Laurent

Lehner, Christian

Lehner, Ben

Lei, Elissa

Lemos, Bernardo

Lenormand, Thomas

Lerit, Dorothy

Lesperance, Danielle

Leu, Jun-Yi

Levit, Georgy S

Lewellyn, Lindsay

Lewis, Zachary

Lewis, Cathryn

Li, Qianyan

Li, Joshua

Li, Huang

Li, Hongjie

Li, Yi-Ju

Li, Xuwen

Li, Pulin

Li, Wenhao

Li, Heng

Li, Jun

Libuda, Diana

Lichten, Michael

Ligoxygakis, Petros

Lin, Yingxin

Link, Nichole

Linksvayer, Timothy

Lion, Sebastien

Lipshitz, Howard

Lisch, Damon

Lister, Ryan

Listgarten, Jennifer

Liu, Chenshu

Liu, Weifang

Liu, Jun

Liu, Ke

Liu, Xiaoqian

Liu, Xiran

Liu, Haoping

Liu, Ke

Llorente, Bertrand

Lloyd, Andrew

Lock, Antonia

Lohmueller, Kirk

Long, Anthony

Long, Jeffrey

Long, Quan

Longo Hollanda de Mello, Pietro

Longworth, Michelle

Lord, Tessa

Loros, Jennifer

Losick, Vicki

Lott, Susan

Lotterhos, Katie

Lovell, Simon

Loverdo, Claude

Lovett, Susan

Luca, Francesca

Lundquist, Erik

Luschnig, Stefan

Lysak, Martin

Ma, Hong

MacAlpine, Jessie

Macdonald, Stuart

Mace, Emma

Machado, Carlos

Macias-Muñoz, Aide

Mackem, Susan

MacLeod, Iona

Macoska, Jill

Maduro, Morris

Maggert, Keith

Magwene, Paul

Mahadevaraju, Sharvani

Makova, Kateryna

Malkova, Anna

Malosetti, Marcos

Mangin, Brigitte

Manichaikul, Ani

Maraia, Richard

Marquardt, Sebastian

Marrec, Loïc

Marti, Emiliano

Martienssen, Rob

Martincorena, Inigo

Martinez-Garcia, Marina

Martinez-Perez, Enrique

Marygold, Steven

Masel, Joanna

Mathieson, Iain

Matunis, Erika

Matute, Daniel

Mayor, Roberto

Mazet, Olivier

McCall, Kimberly

McDonald, Jocelyn

McGaugh, Suzanne

McGlothlin, Joel

McGrath, Patrick

McGuigan, Katrina

McKay, John

McKay, John

McKee, Bruce

McManus, Joel

McStay, Brian

McSteen, Paula

McVey, Mitch

Mead, Timothy

Mech, Aleksandra

Medley, Jeffrey

Medvedev, Paul

Medwig-Kinney, Taylor

Mee, Jonathan

Mefford, Joel

Meiklejohn, Colin

Meirmans, Patrick

Menck, Carlos

Mendez Gonzalez, Ivan

Mercier, Raphae

Merkle, Julie

Messina, Giovanni

Meuwissen, Theo

Meyer, Damon

Meyer, Rhonda

Meyerowitz, Elliot

Miga, Karen

Mikeladze-Dvali, Tamara

Miki, Daisuke

Milano, Carolyn

Millburn, Gillian

Miller, Kenneth

Miller, Rita

Min, Kyung-Jin

Mirkin, Sergei

Mirny, Leonid

Mishra, Abhinava

Mittelsten Scheid, Ortrun

Moazed, Danesh

Moghe, Gaurav

Momany, Michelle

Monroe, J.

Montell, Craig

Montell, Denise

Montgomery, Tai

Morata, Gines

Morgan, Theodore

Morris, Geoffrey

Morrison, Ashby

Morriss, Ginny

Morschhauser, Joachim

Morschhäuser, Joachim

Moses, Alan

Mott, Richard

Mottola, Austin

Moulton, Matthew

Moyle, Leonie

Mueller, Hans-Arno

Mukhopadhyay, Arnab

Müller, Jürg

Mullon, Charles

Munasinghe, Manisha

Munch, Kasper

Muralidhar, Pavitra

Murphy, Helen

Musacchio, Andrea

Musselman, Laura Palanker

Myers, Simon

Nadaf, Javad

Nair, Sudershana

Nakamura, Shuhei

Nakato, Hiroshi

Nambiar, Mridula

Namekawa, Satoshi

Navarro, Arcadi

Navarro, Pau

Navon, Dina

Neale, Matthew

Neher, Richard

Neiman, Aaron

Nelson, Matthew

Nelson, Jonathan

Neuert, Gregor

Neurohr, Gabriel

Nezis, Ioannis

Nghe, Philippe

Nielsen, Dahlia

Ninova, Maria

Niwa, Shinsuke

Noerenberg, Marko

Noonan, James

Nordborg, Magnus

Nordman, Jared

Novembre, John

Novikova, Polina

Nunez, Joaquin

Nyangwara, Vincent

Nystul, Todd

O'Connor, Michael

O'Haren, Thomas

O'Rourke, Eyleen

Oatley, Jon

Ogilvie, Huw

Okae, Hiroaki

Okkema, Peter

Olito, Colin

Oliva, Rafael

Oliver, Brian

Olsen, Anders

Ori-Mckenney, Kassandra

Orr-Weaver, Terry

Ortega-Del Vecchyo, Diego

Ortiz, Rodomiro

Osley, Mary

Osmani, Stephen

Osmond, Matthew

Ostevik, Kate

Otto, Sarah

Oughtred, Rose

Ozdemir, Isa

Ozugergin, Imge

Paaby, Annalise

Page-McCaw, Andrea

Pak, Stephen

Palladino, Francesca

Pallares, Luisa

Palmer, Jeffrey

Pan, Tong

Pan, Chun-Liang

Pandey, Pratima

Pandey, Ashutosh

Panepinto, John

Panzade, Ganesh

Papp, Balazs

Pardo-Manuel de Villena, Fernando

Parikh, Saurin

Park, Hee-Soo

Parkin, Isobel

Parsch, John

Parsons, Todd

Partridge, Linda

Pasero, Phillipe

Pasquinelli, Amy

Patange, Om

Paterson, Andrew

Patterson, Nick

Patton, Elizabeth

Pavinato, Vitor

Pavlov, Youri

Payseur, Bret

Peichel, Catherine

Pellegrini, Matteo

Pellegrino, Mark

Peng, Pei-Chen

Pennings, Pleuni

Pereira-Gómez, Marianoel

Pérez-Rodríguez, Paulino

Perino, Matteo

Peter, Benjamin

Phadnis, Nitin

Phillips, Carolyn

Pierce, Jonathan

Piggott, Beverly

Pikaard, Craig

Pillus, Lorraine

Platt, Alexander

Platt, Frances

Pletcher, Scott

Pocock, Roger

Polechova, Jitka

Polymenis, Michael

Pook, Torsten

Pool, John

Pope, Nathaniel

Port, Fillip

Potter, Christopher

Pourmand, Nader

Preat, Thomas

Preiss, Thomas

Prendergast, James

Price, Jeffrey

Primig, Michael

Pringle, Anne

Program, Peer

Proko, Rinalda

Promislow, Daniel

Pryciak, Peter

Przeworski, Molly

Puchta, Holger

Purugganan, Michael

Qiu, Yinjie

Raftery, Laurel

Ragsdale, Aaron

Rahbari, Raheleh

Rajan, Akhila

Ralph, Peter

Ramachandran, Sohini

Ramakers, Jip

Ramsay, Michele

Rangan, Prashanth

Raz, Erez

Raza, Rabail

Reck-Peterson, Samara

Reed, Laura

Reinhardt, Dieter

Reiser, Michael

Reitz, Diedre

Renicke, Christian

Reusch, Thorsten

Reversade, Bruno

Rhodes, Johanna

Rieder, Leila

Riezman, Howard

Rifkin, Scott

Rios-Barrera, Daniel

Riparbelli, Maria

Rittenhouse, Natalie

Robinson-Thiewes, Sarah

Rocha, Dominique

Rocheleau, Christian

Rockman, Matthew

Rodgers-Melnick, Eli

Rodrigues, Murillo

Rodriguez Lopez, Carlos

Rodríguez Navarro, Susana

Rodriguez-Zas, Sandra

Roeder, Thomas

Rogers, Alicia

Roignant, Jean-Yves

Rokas, Antonis

Romanowski, Andres

Ros, Marian

Rosa, Guilherme

Rosales, Alirio

Rosin, Leah

Ross-Ibarra, Jeffrey

Rothman, Joel

Rotimi, Charles

Rousset, Francois

Roux, Camille

Roze, Denis

Ruan, Jue

Rubin, Seth

Ruden, Douglas

Rueppell, Olav

Rusche, Laura

Rusten, Tor Erik

Ruvinsky, Ilya

Sachdeva, Himani

Sachs, Matthew

Sage, Evelyne

Sahana, Goutam

Sakai, Lynn

Sakamoto, Takahiro

Salminen, Tiina

Saltzman, Arneet

Salz, Helen

Samaranayake, Nilakshi

Samuk, K.

Sanchez-Herrero, Ernesto

Sanchez-Vasquez, Estefania

Sandberg, Rickard

Sandell, Linnea

Santi, Celia

Sanyal, Abhijit

Sapiro, Rossana

Saranga, Yehoshua

Sarinay Cenik, Elif

Sarkies, Peter

Sarsani, Vishal

Sasaki, Akira

Saski, Christopher

Saupe, Sven

Savolainen, Outi

Scally, Aylwyn

Scanlan, Jack

Schacherer, Joseph

Schaeffer, Stephen

Schafer, William

Schartl, Manfred

Schedl, Tim

Schiebel, Elmar

Schierup, Mikkel

Schimenti, John

Schisa, Jennifer

Schlichting, Carl

Schlötterer, Christian

Schnable, Patrick

Schön, Chris-Carolin

Schraiber, Joshua

Schrankel, Catherine

Schrider, Daniel

Schriml, Lynn

Schroeder, Nathan

Schubert, Ingo

Schuldiner, Maya

Schüller, Hans-Joachim

Schumacher, Björn

Schumer, Molly

Schwarzl, Thomas

Schweitzer, Jill

Scornavacca, Celine

Scott, Michael

Scott Chialvo, Clare

Sehgal, Amita

Sekelsky, Jeff

Sekerák, Jiří

Sella, Guy

Sellinger, Thibaut

Selmecki, Anna

Sen, Śaunak

Serbus, Laura

Seroude, Laurent

Setter, Derek

Settles, A.

Seydoux, Geraldine

Seymour, Danelle

Sgro, Carla

Shaffer, Sydney

Shalek, Alex

Sharma Singh, Aditi

Shaver, Amanda

Shaw, Paul

Sheetz, Michael

Shelar, Ashish

Shi, Yongsheng

Shirley, Matthew

Shiroishi, Toshihiko

Shiu, Shin-Han

Shore, David

Shraiman, Boris

Shukla, Diwakar

Sidhwani, Pragya

Siegrist, Sarah

Sieriebriennikov, Bogdan

Silva, Gustavo

Silverman, Neal

Simianer, Henner

Simon, Jeffrey

Simonini, Sara

Simons, Yuval

Simpson, Julie

Singh, Jogender

Sirot, Laura

Siudeja, Kasia

Skeath, James

Skibbens, Robert

Slack, Frank

Slot, Jason

Small, Stephen

Smibert, Craig

Smith, Zack

Smith, Jennifer

Smykal, Petr

Sobel, James

Son, Jae Hak

Song, Michael

Song, Yun

Soroker, Victoria

Soto, Daniela

Spaulding, Emily

Speare, Lauren

Spéder, Pauline

Spence, Jeffrey

Spiliankis, Charalampos

Spradling, Allan

Springer, Nathan

Sreekumar, Lakshmi

Srinivasan, Jagan

Stajich, Jason

Staller, Max

Starz-Gaiano, Michelle

Stavropoulos, Nicholas

Stein, Lincoln

Steinbeck, Christoph

Steinrücken, Matthias

Stern, David

Sternberg, Paul

Stevison, Laurie

Stich, Benjamin

Stirling, Peter

Stitzer, Michelle

Stitzer, Michelle

Stojic, Lovorka

Stollar, Elliott

Storey, John

Stowers, R. Steven

Stranden, Ismo

Stranger, Barbara

Streisfeld, Matthew

Struhl, Gary

Stuart, David

Stukenbrock, Eva

Su, Xiaofeng

Su, Tin Tin

Su, Andrew

Sugden, Lauren

Sugihara, Yu

Sui, Yang

Sullivan, Jack

Sun, Lei

Sundström, Fredrik

Surendranadh, Parvathy

Sutton, Vernon R.

Symington, Lorraine

Szathmary, Eors

Taghert, Paul

Takeishi, Asuka

Takeshita, Norio

Tamkun, John

Tammineni, Eshwar Reddy

Tamura, Koji

Tan, Kaeling

Tanaka, Katsunori

Tanis, Jessica

Tarpy, David

Tavares, Álvaro

Taylor, Peter J.

Taymaz-Nikerel, Hilal

Tempelman, Robert

Temple, Seth

Tenaillon, Maud

Tennessen, Jason

Terhorst, Jonathan

Thakur, Rajan

Thompson, Ken

Thon, Genevieve

Thornton, Kevin

Thornton, Joe

Thorpe, Peter

Thummel, Carl

Tian, Dacheng

Timpson, Nicholas

Tishkoff, Sarah

Todd, Richard

Todesco, Marco

Tootle, Tina

Toro, Sabrina

Torres-Machorro, Ana

Tosetto, Louise

Tourrette, Elise

Tovar, Adelaide

Townsend, Jeffrey

Trachtulec, Zdenek

Trapnell, Cole

Treisman, Jessica

TRUE, Heather

Trynka, Gosia

Tu, Benjamin

Tuller, Tamir

Tung, Chih Kuan

Turcotte, Carolyn

Turelli, Michael

Turner, Glenn

Tuschl, Karin

Tyers, Mike

Udall, Joshua

Uecker, Hildegard

Ueda, Hiroki

Ulitski, Igor

Ünal, Elçin

Unckless, Robert

Underwood, Charles

Updike, Dustin

Urban, Martin

Valdar, William

Van Dam, Sipko

Van der Burght, Servaas

Van der Werf, Julius

Van der Werf, Julius

Van Steensel, Bas

Van Werven, Folkert

Van Wolfswinkel, Josien

Vande Velde, Christine

Vandebergh, Marijne

Varona, Luis

Varshney, Neha

Vartiainen, Maria

Vázquez, Ángel Vizoso

Vázquez, Ángel Vizoso

Véber, Amandine

Veller, Carl

Venkatachalam, Kartik

Venkatachalam, Vivek

Venken, Koen

Vershon, Andrew

Vicoso, Beatriz

Vitalis, Renaud

Vize, Peter

Vogan, Aaron

Vogel, Bruce

Voight, Ben

Vokes, Steven

Vollbrecht, Erik

Voss-Fels, Kai

Vosshall, Leslie

Wagner, Maggie

Wahl, Lindi

Wain, Louise

Wakimoto, Barbara

Walhout, Albertha

Walker, Marquis

Walker, Amy

Walker, David

Wallace, Jason

Walsh, J.

Wang, Miaoyang

Wang, Zuoheng

Wang, Jianmin

Wang, Liguo

Wang, Yuan

Wang, Ming-Bo

Wang, Xu

Wang, Yu-Feng

Wang, Zonghua

Wani, Khursheed

Ward, Jordan

Waterston, Robert

Watts, Jennifer

Waugh, Robbie

Weaver, Lesley

Wehman, Ann

Wei, Kevin

Weissman, Daniel

Welch, John

Weng, Xiaoyu

Wessel, Gary

Westerfield, Monte

Westerfield, Monte

Whibley, Annabel

Whipple, Clinton

White, Benjamin

White, Michael

White, Kristin

Whitehead, Andrew

Whiteway, Malcolm

Whitlock, Michael

Whittaker, John

Wientjes, Yvonne

Wiessman, Daniel

Wiggans, George

Wigler, Michael

Wignall, Sarah

Wijesekera, Thilini

Wilke, Claus

Wilkins, Adam

Williams, Robert

Willighagen, Egon

Wilson, Stephen

Wilson, Thomas

Wilton, Peter

Winston, Fred

Wise, Roger

Wittkopp, Patricia

Wolfe, Kenneth

Wolfe, Marnin

Wolfner, Mariana

Woodhouse, Margaret

Woods, Daniel

Wright, Dominic

Wu, Xiaohua

Wu, Ting

Wu, Michael

Wu, Mark

Wu, Si

Wurtele, Hugo

Xi, Rongwen

Xiang, Ruidong

Xing, Yi

Xu, Hong

Xu, Jianping

Xu, Shizhong

Xu, Shizhong

Xue, Angli

Yadav, Vikas

Yagoobi, Sedigheh

Yamamoto, Shinya

Yamanaka, Naoki

Yamashita, Yukiko

Yamashita, Yukiko

Yang, Tianzhong

Yang, Hua

Yang, Chin Jian

Yang, Yungui

Yanovsky, Marcelo

Yanowitz, Judith

Yarikipati, Prathibha

Yates, Andrew

Yeaman, Sam

Yemini, Eviatar

Yengo, Loic

Yogev, Shaul

Yu, Miao

Yu, Jae-Hyuk

Yuan, Kai

Yuh Chwen Lee, Grace

Zaheer, Tean

Zarate-Potes, Alejandra

Zeng, Jian

Zeng, Lei

Zerges, William

Zhang, Xinjun

Zhang, Yiliang

Zhang, Jianzhi

Zhao, Keji

Zhao, Meixia

Zhao, Fangqing

Zhao, Li

Zheng, Chaozhi

Zheng, Hou-Feng

Zhimulev, Igor

Zhong, Yi

Zhou, Geyu

Zhou, Wei

Zhou, Yun

Zhou, Rui

Zhuang, Xuan

Zi, Zhike

Zilberman, Daniel

Ziv, Elad

Zook, Justin

Zorn, Aaron

Zou, Zhengting

